# (Multiscale) Cross-Entropy Methods: A Review

**DOI:** 10.3390/e22010045

**Published:** 2019-12-29

**Authors:** Antoine Jamin, Anne Humeau-Heurtier

**Affiliations:** 1 COTTOS Médical, Allée du 9 novembre 1989, 49240 Avrillé, France; 2 LARIS – Laboratoire Angevin de Recherche en Ingénierie des Systèmes, Univesity Angers, 62 avenue Notre-Dame du Lac, 49000 Angers, France; anne.humeau@univ-angers.fr

**Keywords:** cross-entropy, multiscale cross-entropy, asynchrony, complexity, coupling, cross-sample entropy, cross-approximate entropy, cross-distribution entropy, cross-fuzzy entropy, cross-conditional entropy

## Abstract

Cross-entropy was introduced in 1996 to quantify the degree of asynchronism between two time series. In 2009, a multiscale cross-entropy measure was proposed to analyze the dynamical characteristics of the coupling behavior between two sequences on multiple scales. Since their introductions, many improvements and other methods have been developed. In this review we offer a state-of-the-art on cross-entropy measures and their multiscale approaches.

## 1. Introduction

To quantify the asynchronism between two time series, Pincus and Singer have adapted the approximate entropy algorithm to a cross-approximate entropy (cross-ApEn) method [1]. Then, other cross-entropy methods—that improve the cross-ApEn—have been developed [2,3,4,5,6,7]. Furthermore, additional cross-entropy methods have been introduced to quantify the degree of coupling between two signals, or the complexity between two cross-sequences [8,9,10]. Cross-entropy methods have recently been used in different research fields, including medicine [5,11,12], mechanics [13], and finance [7,10].

The multiscale approach of entropy measures was proposed by Costa et al. in 2002 to analyze the complexity of a time series [14]. In 2009, Yan et al. proposed a multiscale approach for cross-entropy methods to quantify the dynamical characteristics of coupling behavior between two sequences on multiple scale factors [15]. Then, other multiscale procedures have been published with different cross-entropy methods [16,17]. Multiscale cross-entropy methods have recently been used in different research fields, including medicine [18,19,20,21], finance [6,9], civil engineering [22], and the environment [23].

Cross-entropy methods and their multiscale approaches are used to obtain information on the possible relationship between two time series. For example, Wei et al. applied percussion entropy to the amplitude of digital volume pulse signals and changes in R-R intervals of successive cardiac cycles for assessing baroreflex sensitivity [18]. Results showed that the method is able to identify the markers of diabetes by the nonlinear coupling behavior of the two cardiovascular time series. Moreover, Zhu and Song computed cross-fuzzy entropy on a vibration time series to assess the bearing performance degradation process of motor [13]. Results showed that the method detects trend for bearing degradation process over the whole lifetime. In addition, Wang et al. applied multiscale cross-trend sample entropy to analyze the asynchrony between air quality impact factors (fine particulate matters, nitrogen dioxide, …), and air quality index (AQI) in different regions of China [23]. Results showed that the degree of synchrony between fine particulate matter and AQI is higher than the other air quality impact factor which reveals that fine particulate matter has become the main source of air pollution in China.

Our paper presents a state-of-the-art in three sections: First, the cross-entropy methods are introduced. We detail, in the second section, different multiscale procedures. A multiscale cross-entropy generalization is presented and other specific multiscale cross-entropy algorithms are proposed in the third section.

## 2. Cross-Entropy Methods

In this section, we classify cross-entropy methods according to their entropy measures: Cross-approximate entropy, cross-sample entropy, and cross-distribution entropy. Other methods that use different cross-entropy-based measures are also detailed. Table 1 shows the twelve measures that are detailed in this section.

### 2.1. Cross-Approximate Entropy-Based Measures

#### 2.1.1. Cross-Approximate Entropy

Cross-approximate entropy (cross-ApEn), introduced by Pincus and Singer [1], allows to quantify asynchrony between two time series. For two vectors u and v of length *N*, cross-ApEn is computed as:

(1) $$\mathrm{cross}-\mathrm{ApEn}(m,r,N)\left(v||u\right)=\Phi^{m}\left(r\right)\left(v||u\right)-\Phi^{m+1}\left(r\right)\left(v||u\right),$$

where $\Phi^{m}\left(r\right)\left(v||u\right)=\frac{1}{N-m+1}\sum_{i=1}^{N-m+1}\log C_{i}^{m}\left(r\right)\left(v||u\right)$ and $C_{i}^{m}\left(r\right)\left(v||u\right)$ is the number of sequences, of *m* consecutive points, of u that are approximately (within a resolution *r*) the same as sequences, of the same length, of v. One major dawback of this approach is that $C_{i}^{m}\left(r\right)\left(v||u\right)$ should not be equal to zero. This is why cross-ApEn is not really adapted for a short time series. Furthermore, it is direction-dependent because often $\Phi^{m}\left(r\right)\left(v||u\right)$ is generally not equal to its direction conjugate $\Phi^{m}\left(r\right)\left(u||v\right)$ [2]. The value of cross-ApEn computed from two signals can be interpreted as a degree of synchrony or mutual relationship.

#### 2.1.2. Binarized Cross-Approximate Entropy

Binarized cross-approximate entropy (XBinEn), introduced by Škorić et al. [5] in 2017, is an evolution of cross-ApEn to quantify the similarity between two time series. It has the advantage of being faster than cross-ApEn. XBinEn encodes a time series divided into vectors of length *m*. For two vectors u and v of length *N*, the XBinEn algorithm follows these six steps:

1. Binary encoding series are obtained as:
  (2) $$x_{i}=\left\{\begin{array}{cc} 0 & \mathrm{if}\;u_{i+1}-u_{i}⩽0\\ 1 & \mathrm{if}\;u_{i+1}-u_{i}>0 \end{array}\right.,\;y_{i}=\left\{\begin{array}{cc} 0 & \mathrm{if}\;v_{i+1}-v_{i}⩽0\\ 1 & \mathrm{if}\;v_{i+1}-v_{i}>0 \end{array}\right.,$$
  where i=1,2,…,N−1, $x_{i}\in X_{m}^{\left(i\right)}=\left[x_{i},x_{i+t},\ldots ,x_{i+(m-1)t}\right]$, and $y_{i}\in Y_{m}^{\left(i\right)}=\left[y_{i},y_{i+t},\ldots ,y_{i+(m-1)t}\right]$.
  The time lag *t* allows a vector decorrelation to be performed;
2. Vector histograms $N_{X}^{\left(m\right)}\left(k\right)$ and $N_{Y}^{\left(m\right)}\left(n\right)$ are computed as:
  (3) $$N_{X}^{\left(m\right)}\left(k\right)=\sum_{i=1}^{N-(m-1)t}I\left\{\sum_{l=0}^{m-1}x_{i+l\cdot t}\times 2^{l}=k\right\},\;N_{Y}^{\left(m\right)}\left(n\right)=\sum_{j=1}^{N-(m-1)t}I\left\{\sum_{l=0}^{m-1}y_{j+l\cdot t}\times 2^{l}=n\right\},$$
  where $k,n=0,1,\ldots ,2^{m}-1$, and I{·} is a function that is equal to 1 if the indicated condition is fulfilled;
3. The probability mass functions are obtained as:
  (4) $$P_{X}^{\left(m\right)}\left(k\right)=\frac{N_{X}^{\left(m\right)}\left(k\right)}{N-(m-1)t},\;P_{Y}^{\left(m\right)}\left(n\right)=\frac{N_{Y}^{\left(m\right)}\left(n\right)}{N-(m-1)t},$$
  where $k,n=0,1,\ldots ,2^{m}-1$;
4. A distance measure is applied:
  (5) $$d\left(X_{m}^{\left(i\right)},Y_{m}^{\left(j\right)}\right)=\sum_{k=0}^{m-1}I\left\{x_{i+k\cdot t}\neq y_{j+k\cdot t}\right\},$$
  where i,j=1,…,N−(m−1)t;
5. The probability $p_{k}^{m}\left(r\right)$ that a vector is within the distance *r* from a particular vector is estimated:
  (6) $$p_{k}^{m}\left(r\right)=\mathrm{Pr}\left\{d\left(X_{m}^{\left(k\right)},Y_{m}\right)⩽r\right\};$$
6. XBinEn is finally obtained as:
  (7) $$\mathrm{XBinEn}\left(m,r,N,t\right)=\Phi^{\left(m\right)}\left(r,N,t\right)-\Phi^{\left(m+1\right)}\left(r,N,t\right),$$
  where $\Phi^{\left(m\right)}\left(r,N,t\right)=\sum_{k=0}^{2^{m}-1}P_{X}^{\left(m\right)}\left(k\right)\cdot \ln\left(p_{k}^{m}\left(r\right)\right)$.

This method gives almost the same results as cross-ApEn for a non-short time series. However, it is computationally more efficient than cross-ApEn. Its main disadvantage is that it cannot identify small signal changes. XBinEn is adapted to environments where processor resources and energy are limited but it is not a substitute to cross-ApEn [5]. It is proposed when the cross-ApEn procedure cannot be applied. The value of XBinEn computed from two signals can be interpreted as a degree of relationship between a related pair of time series.

### 2.2. Cross-Sample Entropy-Based Measures

#### 2.2.1. Cross-Sample Entropy

Cross-sample entropy (cross-SampEn) quantifies the degree of asynchronism of two time series. This method was introduced by Richman and Moorman in 2000 to improve the cross-ApEn limitations (see Section 2.1.1) [2]. Cross-SampEn is a conditional probability measure that quantifies the probability that a sequence of *m* consecutive points (called sample) of a time series u—that matches another sequence of the same length of another time series v—will still match the other sequence when their length is increased by one sample (m+1). For two vectors u and v, cross-SampEn is computed as:

(8) $$\mathrm{cross}-\mathrm{SampEn}\left(m,r,N\right)\left(v||u\right)=-\ln\frac{A^{m}\left(r\right)\left(v||u\right)}{B^{m}\left(r\right)\left(v||u\right)},$$

where *m* is the sample length, *N* is the vectors (u and v) length, $A^{m}\left(r\right)\left(v||u\right)$ and $B^{m}\left(r\right)\left(v||u\right)$ are, respectively, the probability that a sequence of u and a sequence of v will match for m+1 and *m* points (within a tolerance *r*).

For two time series u and v of length *N*, cross-SampEn can also be described as:

(9) $$\mathrm{cross}-\mathrm{SampEn}\left(u,v,m,r,N\right)=-\ln\frac{n^{\left(m+1\right)}}{n^{\left(m\right)}},$$

where $n^{\left(m\right)}$ represents the total number of sequences of *m* consecutive points of u that match with other sequences of *m* consecutive points of v.

The main difference between cross-ApEn and cross-SampEn is that cross-SampEn shows relative consistency whereas cross-ApEn does not. Unlike cross-ApEn, cross-SampEn is not direction-dependent. However, cross-SampEn generates, sometimes, undefined values for short time series. The value of cross-SampEn computed from two time series can be interpreted as a measure of similarity of the two time series.

#### 2.2.2. Modified Cross-Sample Entropy

Modified cross-sample entropy (mCSE), introduced by Yin and Shang in 2015, has been developed to detect the asynchrony of a financial time series [4]. Inspired by the generalized sample entropy, proposed by Silva and Murta, Jr. [25], the authors proposed to adapt this method to cross-SampEn. The method combines cross-SampEn and nonadditive statistics. For two vectors u and v of length *N*, mCSE is computed as:

(10) $$\mathrm{mCSE}\left(m,r,N\right)=-\log_{q}\frac{\sum_{i=1}^{N-m}n_{i}^{\left(m+1\right)}}{\sum_{i=1}^{N-m}n_{i}^{\left(m\right)}},$$

where *m* is the sample length, *q* is the entropic index, and $n_{i}^{\left(m\right)}$ is the number of times that the distance between vectors $y_{m}=\left\{v\left(i\right),v\left(i+1\right),\ldots ,v\left(i+m-1\right):1⩽i⩽N-m+1\right\}$ and $x_{m}=\left\{u\left(i\right),u\left(i+1\right),\ldots ,u\left(i+m-1\right):1⩽i⩽N-m+1\right\}$ is less than or equal to the tolerance *r*. The distance is calculated with $d\left(x_{m}\left(i\right),y_{m}\left(i\right)\right)=\max\left\{|u\left(i+k\right)-v\left(j+k\right)|:0⩽k⩽m-1\right\}$.

The value of mCSE computed from two time series can be interpreted as a degree of synchrony between the two time series and it can illustrate some intrinsic relations between the two time series.

#### 2.2.3. Modified Cross-Sample Entropy Based on Symbolic Representation and Similarity

Modified cross-sample entropy based on symbolic representation and similarity (MCSEBSS), introduced by Wu et al. in 2018, has been developed to quantify the degree of asynchrony of two financial time series with various trends (stock markets from different areas [6]). In comparison with cross-SampEn, this method reduces the probability of including undefined entropies and it is more robust to noise. For two vectors u and v of length *N*, MCSEBSS is computed as:

(11) $$\mathrm{MCSEBSS}\left(u,v,m,r,N\right)=-\ln\frac{n^{\left(m+1\right)}}{n^{\left(m\right)}},$$

where *m* is the sample length and $n^{\left(m\right)}$ is the number of template matches by comparing $s(u_{m}\left(i\right),v_{m}\left(j\right))$ and *r*. For $u_{m}=\left\{u\left(i+k\right)\right\}$ and $v_{m}=\left\{v\left(i+k\right)\right\}$ (0⩽k⩽m−1 and 1⩽i⩽N−m), the similarity function $s(u_{m}\left(i\right),v_{m}\left(j\right))$ is calculated as:

(12) $$s\left(u_{m}\left(i\right),v_{m}\left(j\right)\right)=\frac{\#\;\mathrm{of}\;\mathrm{in}\;\mathrm{count}(i,j)}{m},\;1⩽i,j⩽N-m,$$

where count(i,j) is obtained by the function *f* defined as:

(13) $$f=\left\{\begin{array}{cc} 1 & \mathrm{if}\;u_{m}\left(i+k\right)=v_{m}\left(j+k\right)\\ 0 & \mathrm{if}\;u_{m}\left(i+k\right)\neq v_{m}\left(j+k\right) \end{array}\right.,\;0⩽k⩽m-1.$$

The parameter *r* must be fixed between $\frac{m-n}{m+1}$ and $\frac{m-n}{m}$, where *n* is the maximum number of zeros obtained with count(i,j) to consider u and v similar.

The value of MCSEBSS computed from two time series can be interpreted as a degree of asynchrony of the two time series. A low cross-entropy value indicates a strong synchrony between two signals.

#### 2.2.4. Kronecker-Delta-Based Cross-Sample Entropy

The Kronecker-delta-based cross-sample entropy (KCSE), introduced by He et al. in 2018, has been developed to define the dissimilarity between two time series [7]. KCSE is based on the Kronecker-delta function $\delta_{x,y}$ that returns 1 if two variables are equal and 0 otherwise. For two vectors u and v of length *N*, KCSE is calculated as:

(14) $$\mathrm{KCSE}\left(m\right)=-\ln\frac{B^{m+1}}{B^{m}},$$

where $B^{m}=\frac{\sum_{i=1}^{N-m+1}{\mathrm{KrD}}_{u_{m}\left(i\right),v_{m}\left(i\right)}}{N-m+1}$ and $B^{m+1}=\frac{\sum_{i=1}^{N-m}{\mathrm{KrD}}_{u_{m+1}\left(i\right),v_{m+1}\left(i\right)}}{N-m}$. The dissimilarity, between $u_{m}\left(i\right)=\left[u\left(i\right),u\left(i+1\right),\ldots ,u\left(i+m-1\right)\right]$ and $v_{m}\left(i\right)=\left[v\left(i\right),v\left(i+1\right),\ldots ,v\left(i+m-1\right)\right]$, is calculated as:

(15) $${\mathrm{KrD}}_{u_{m}\left(i\right),v_{m}\left(i\right)}=\frac{\delta_{u_{m}\left(i\right),v_{m}\left(i\right)}+\delta_{u_{m}\left(i+1\right),v_{m}\left(i+1\right)}+\cdots +\delta_{u_{m}\left(i+m-1\right),v_{m}\left(i+m-1\right)}}{n}.$$

Authors show that KCSE is better to classify financial data than multidimensional scaling based on the Chebyshev distance method [7]. The value of KSCE computed from two time series can be interpreted as a degree of irregularity between the two time series.

#### 2.2.5. Permutation-Based Cross-Sample Entropy

The permutation-based cross-sample entropy (PCSE), introduced by He et al. in 2018, is quite similar to KCSE (see Section 2.2.4) [7]. A permutation step has only been added. For two vectors u and v of length *N*, PCSE is calculated as:

(16) $$\mathrm{PCSE}\left(m\right)=-\ln\frac{B^{m+1}}{B^{m}},$$

where $B^{m}=\frac{\sum_{i=1}^{N-m+1}{\mathrm{KrD}}_{{\mathrm{permuX}}_{m}\left(i\right),{\mathrm{permuY}}_{m}\left(i\right)}}{N-m+1}$ and $B^{m+1}=\frac{\sum_{i=1}^{N-m}{\mathrm{KrD}}_{{\mathrm{permuX}}_{m+1}\left(i\right),{\mathrm{permuY}}_{m+1}\left(i\right)}}{N-m}$. The KrD function is defined in Section 2.2.4. The two vectors ${\mathrm{permuX}}_{m}\left(i\right)$ and ${\mathrm{permuY}}_{m}\left(i\right)$ are obtained by a permutation algorithm defined with the permutation entropy [26]. The Video S1 shows an example of a permutation algorithm.

PCSE shows better results than KCSE for synthetic data (ARFIMA model) [7]. However, the two approaches give the same results for financial data [7]. Authors show that KCSE is better to classify financial data than multidimensional scaling based on the Chebyshev distance method [7]. The value of PCSE computed from two time series can be interpreted as degree of irregularity between the two time series.

#### 2.2.6. Cross-Trend Sample Entropy

Inspired by MCSEBSS (see Section 2.2.3), Wng et al. developed the cross-trend sample entropy (CTSE) to quantify the synchronism between two time series with strong trends [23]. For two time series u and v of length *N*, CTSE is calculated with the following four steps algorithm:

1. The two time series are symbolized as:
  (17) $$U\left(j\right)=\left\{\begin{array}{cc} 1 & \mathrm{if}\;\tilde{u}\left(j\right)>u\left(j\right)\\ 0 & \mathrm{otherwise} \end{array}\right.,\;V\left(j\right)=\left\{\begin{array}{cc} 1 & \mathrm{if}\;\tilde{v}\left(j\right)>v\left(j\right)\\ 0 & \mathrm{otherwise} \end{array}\right.,\;1⩽j⩽N,$$
  where $\tilde{u}$ and $\tilde{v}$ are, respectively, the trend of u and v obtained by polynomial fitting (linear, quadratic or higher order).
2. The template vectors $u_{m}$ and $v_{m}$ are constructed as:
  (18) $$u_{m}\left(i\right)=\left\{U\left(i+k\right)\right\},\;v_{m}\left(i\right)=\left\{V\left(i+k\right)\right\},$$
  where 0⩽k⩽m−1 and 1⩽i⩽N−m.
3. The similarity between $x_{m}\left(i\right)$ and $y_{m}\left(i\right)$ is calculated as:
  (19) $$d\left(x_{m}\left(i\right),y_{m}\left(i\right)\right)=\frac{\#\;\mathrm{of}\;1\;\mathrm{in}\;C_{m}\left(i\right)}{m},\;1⩽i⩽N-m,$$
  where the *i*-th symbol vector $C_{m}$ is determined with *f*, a symbolic function between two template vectors $u_{m}$ and $v_{m}$, as:
  (20) $$f=\left\{\begin{array}{cc} 1 & \mathrm{if}\;u_{m}\left(i+k\right)=v_{m}\left(i+k\right)\\ 0 & \mathrm{otherwise} \end{array}\right.,\;0⩽k⩽m-1.$$
4. CTSE is finally computed as:
  (21) $$\mathrm{CTSE}\left(u,v,r,N\right)=-\ln\frac{n^{\left(m+1\right)}}{n^{\left(m\right)}},$$
  where $n^{\left(m\right)}$ is obtained by comparing $d(x_{m}\left(i\right),y_{m}\left(j\right))$ within a tolerance *r* for 1⩽i⩽N−m.

CTSE has two advantages over MCSEBSS: It is more sensitive to the difference of dynamical characteristic between two signals, and it works well with signals with trends (linear, quadratic, cubic, and sinusoidal) [23]. The value of CTSE computed from two time series can be interpreted as an indicator of dynamical structure regarding the two time series with potential trends.

### 2.3. Cross-Distribution Entropy-Based Measures

#### 2.3.1. Cross-Distribution Entropy

In 2018, Wang and Shang introduced the cross-distribution entropy (cross-DistEn) to quantify the complexity between two cross-sequences [9]. To generalize the standard statistical mechanics, the authors replaced the standard distribution entropy (DistEn) based on Shannon entropy by DistEn based on Tsallis entropy [9]. The authors showed that cross-DistEn better illustrates the relationships between two vectors than cross-SampEn does [9]. For two times series u and v of length *N* cross-DistEn follow these four steps:

1. The state-space is reconstructed by building (N−m+1) vectors X(i) and Y(i) with X(i)={u(i),u(i+1),…,u(i+m−1)}, 1≤i≤N−m, and Y(j)={v(j),v(j+1),…,v(j+m−1)}, 1≤j≤N−m. *m* is the intended size of the vectors X(i) and Y(i);
2. The distance matrix is built by defining the distance matrix $D=\{d_{i,j}\}$ with $d_{i,j}$ being the Chebyshev distance between the vectors X(i) and Y(j) defined as:
  (22) $$d_{ij}=\max\{|u_{i+k}^{\left(\tau \right)}-v_{j+k}^{\tau }\left|,0⩽k⩽m-1\right\};$$
3. The probability density is estimated by computing the empirical probability density function of the matrix D by applying the histogram approach. If the histogram has *M* bins, the probability of each bin will be $P_{t}$ with 1≤t≤M;
4. The cross-distribution entropy based on the Tsallis entropy is computed as:
  (23) $$crossDistEn\left(u,v\right)=\frac{1}{\ln\left(a\right)}\frac{1}{q-1}\left(1-\sum_{t=1}^{M}P_{t}^{q}\right),$$
  where *q* is the order of the Tsallis entropy and *a* the logarithm base of the entropy computation.

The main advantage of cross-DistEn is that it is adapted for short time series. With financial data, cross-DistEn illustrates better the relationship between signals than cross-SampEn [9]. The value of cross-DistEn computed from two time series can be interpreted as a degree of linkage of the two time series.

#### 2.3.2. Permutation Cross-Distribution Entropy

The permutation cross-distribution entropy (PCDE), introduced by He et al. in 2019, is a variant of cross-DistEn (see Section 2.3.1) [10]. The permutation allows to characterize fluctuations and prevents the impact of spatial distances on results. The PCDE algorithm is the same as the one of cross-DistEn, detailed in Section 2.3.1. However, an additional step is added before step 2 to permute X(i) and Y(j) with the permutation algorithm mentioned in Section 2.2.5. The distance matrix is therefore constructed with the permuted vectors. The value of PCDE computed from two time series can be interpreted as a degree of dissimilarity between the two time series.

### 2.4. Other Cross-Entropy-Based Measures

#### 2.4.1. Cross-Conditional Entropy

Cross-conditional entropy (CCE), introduced by Porta et al. in 1919, quantifies the degree of coupling between two signals [8]. A corrected conditional entropy has been introduced to improve the approximate entropy that suffers from limitations when a finite number of sample is considered [27]. CCE is an adaptation of the corrected conditional entropy. For two signals u={u(i),i=1,…,N} and v={v(i),i=1,…,N}, CCE is computed as:

(24) $${\mathrm{CCE}}_{v/u}\left(L\right)=-\sum_{L-1}p\left(u_{L-1}\right)\sum_{i/L-1}p\left(v\left(i\right)/u_{L-1}\right)\times \log p(v\left(i\right)/u_{L-1}),$$

where *L* is the length of the pattern extracted to be compared, $p(u_{L-1})$ is the joint probability of the pattern $u_{L-1}\left(i\right)=\left(u\left(i\right),u_{L-1}\left(i-1\right)\right)$, and $p(v\left(i\right)/u_{L-1})$ is the probability of the sample v(i) given the pattern $u_{L-1}\left(i\right)$. If a mixed pattern, composed by L−1 samples, of u and v: $\left(v\left(i\right),u\left(i\right),\ldots ,u\left(i-L+2\right)\right)=\left(v\left(i\right),u_{L-1}\right)$, is defined and with the Shannon entropy $E\left(u_{L}\right)=-\sum_{L}p\left(u_{L}\right)\log p(u_{L})$, CCE can also be described as:

(25) $${\mathrm{CCE}}_{v/u}\left(L\right)=E\left(v\left(i\right),u_{L-1}\right)-E\left(u_{L-1}\right).$$

For a limited amount of samples, the approximation of CCE always decreases to zero while increasing L. To solve this problem, a modification has been introduced as:

(26) $${\mathrm{CCE}}_{v/u}\left(L\right)=\hat{{\mathrm{CCE}}_{v/u}}\left(L\right)+{\mathrm{perc}}_{v/u}\left(L\right)\times \hat{E}\left(v\right),$$

where ${\mathrm{perc}}_{v/u}$ is the ratio of mixed patterns found only once over the total number of mixed patterns, $\hat{{\mathrm{CCE}}_{v/u}}\left(L\right)$ and $\hat{E}\left(v\right)$ are, respectively, the estimates of the ${\mathrm{CCE}}_{v/u}\left(L\right)$ and E(v) based on the considered limited dataset.

CCE can be defined as a measure of unpredictability of one signal when the second is observed because it quantifies the amount of information carried by one signal which cannot be derived from the other. It is not fully a measure of synchronization. The main disadvantage of CCE is that it is not totally adapted for short time series.

#### 2.4.2. Cross-Fuzzy Entropy

Cross-fuzzy entropy (C-FuzzyEn), introduced by by Xie et al. in 2010 [3], is an adaptation of fuzzy entropy, introduced by Chen et al. [28], that quantifies the synchrony or similarity of patterns between two signals [3]. C-FuzzyEn is an improvement of cross-SampEn that is more adapted for short time series and more robust to noise. For two times series u and v of length *N*, C-FuzzyEn is obtained with the following three steps algorithm:

1. The distance $d_{ij}^{m}$ between $X_{i}^{m}$ and $Y_{j}^{m}$ is computed as:
  (27) $$d_{ij}^{m}=d\left[X_{i}^{m},Y_{j}^{m}\right]=\max_{k\in (0,m-1)}\left|u\left(i+k\right)-\bar{u}\left(i\right)-v\left(j+k\right)-\bar{v}\left(i\right)\right|,$$
  where *m* is the number of consecutive data to compare, $X_{i}^{m}=\left\{u\left(i\right),u\left(i+1\right),\ldots ,u\left(i+m-1\right)\right\}-\bar{u}\left(i\right)$, and $Y_{j}^{m}=\left\{v\left(i\right),v\left(i+1\right),\ldots ,u\left(v+m-1\right)\right\}-\bar{v}\left(i\right)$. $\bar{u}\left(i\right)$ and $\bar{v}\left(i\right)$ are calculated as: $\bar{u}\left(i\right)=\frac{1}{m}\sum_{l=0}^{m-1}u\left(i+l\right)$, and $\bar{v}\left(i\right)=\frac{1}{m}\sum_{l=0}^{m-1}v\left(i+l\right)$;
2. The synchrony or similarity degree $D_{ij}^{m}$ is computed as: $D_{ij}^{m}=\mu \left(d_{ij}^{m},n,r\right)$, where $\mu (d_{ij}^{m},n,r)$ is the fuzzy function obtained as:
  (28) $$\mu \left(d_{ij}^{m},n,r\right)=\exp-\frac{{\left(d_{ij}^{m}\right)}^{n}}{r},$$
  where *r* and *n* determine the width and the gradient of the boundary of the exponential function, respectively;
3. Finally, C-FuzzyEn is computed as:
  (29) $$C-\mathrm{FuzzyEn}\left(m,n,r\right)=\ln\Phi^{m}-\ln\Phi^{m+1},$$
  where $\Phi^{m}=\frac{1}{N-m}\sum_{i=1}^{N-m}\left(\frac{1}{N-m}\sum_{j=1}^{N-m}D_{ij}^{m}\right)$, and $\Phi^{m+1}=\frac{1}{N-m}\sum_{i=1}^{N-m}\left(\frac{1}{N-m}\sum_{j=1}^{N-m}D_{ij}^{m+1}\right)$.

The value of C-FuzzyEn computed from two time series can be interpreted as the synchronicity of patterns.

#### 2.4.3. Joint-Permutation Entropy

Joint permutation entropy (JPE), introduced by Yin et al. in 2019, quantifies the synchronism between two time series. It is based on permutation entropy that consists of comparing neighboring values of each point and mapping them to ordinal patterns to quantify the complexity of a signal [26]. For two signals u and v, JPE is computed as the Shannon entropy of the d!×d! distinct motif combinations $\left\{\left(\pi_{i}^{d,t},\pi_{j}^{d,t}\right)\right\}$:

(30) $$\mathrm{JPE}\left(d,t\right)=-\sum_{i,j:(\pi_{i}^{d,t},\pi_{j}^{d,t})}p\left(\pi_{i}^{d,t},\pi_{j}^{d,t}\right)\cdot \ln p\left(\pi_{i}^{d,t},\pi_{j}^{d,t}\right),$$

where *d* is the embedded dimension and $p(\pi_{i}^{d},\pi_{j}^{d})$ is the joint probability of $\left\{\left(\pi_{i}^{d,t},\pi_{j}^{d,t}\right)\right\}$ appearing in the $X_{l}^{d,t}=\left\{u_{l},u_{l+t},\ldots ,u_{l+(d-1)t}\right\}$ and $Y_{l}^{d,t}=\left\{v_{l},v_{l+t},\ldots ,v_{l+(d-1)t}\right\}$ and it is defined as:

(31) $$p\left(\pi_{i}^{d,t},\pi_{j}^{d,t}\right)=\frac{||l:l⩽T,\mathrm{type}\left(X_{l}^{d,t},Y_{l}^{d,t}\right)=\left(\pi_{i}^{d,t},\pi_{j}^{d,t}\right)\left|\right|}{T},$$

where T=N−(d−1)t, type(·) corresponds to the map from pattern space to symbol space, and ||·|| corresponds to the cardinality of a set.

The main advantages of JPE are the simplicity, the robustness, and the low computational cost. The value of JPE computed from two time series can be interpreted as a degree of correlation between the two time series [29].

## 3. Multiscale Procedures

To study entropy or cross-entropy measures of time series across scales, a multiscale procedure can be used. In this part we detail, in chronological order, three multiscale methods: The coarse-grained, the time-shift, and the composite coarse-grained approaches.

### 3.1. Coarse-Graining Procedure

**Figure 1:**
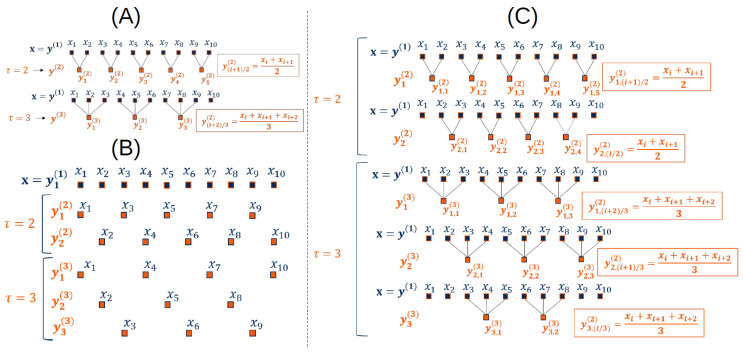
Examples of multiscale procedures for the ten first points of a time series x. (**A**) represents the coarse-graining procedure (modified from [34]), (**B**) shows the time-shift procedure, and (**C**) illustrates the composite coarse-graining procedure (modified from [35]).

In 2002 Costa et al. introduced the coarse-graining procedure to analyze the complexity, defined by the analysis of the irregularity through scale factors [14]. This method is an improvement, more adapted for a biological time series, of the coarse-graining procedure introduced by Zhang [30]. This procedure has been used in multiscale entropy and cross-entropy methods [6,9,15,20,31,32,33]. For each scale factor, this procedure derives a set of vectors illustrating the system dynamics. For a monovariate discrete signal x of length *N*, the coarse-grained time series $y^{\left(\tau \right)}$ is calculated as:

(32) $$y_{j}^{\left(\tau \right)}=\frac{1}{\tau }\sum_{i=(j-1)\tau +1}^{j\tau }x_{i},$$

where τ is the scale factor and $1⩽j⩽\frac{N}{\tau }$. The length of the coarse-grained vector is $\frac{N}{\tau }$. An example of coarse-graining procedure is presented in Figure 1A.

### 3.2. Time-Shift Procedure

As for the coarse-grained procedure, the time-shift procedure is used to decompose a signal through different scale factors and to perform a multiscale analysis. While coarse-graining procedure uses the averaging of time series on several interval scales, the time-shift procedure applies time shifting in time series. The main disadvantage of a coarse-graining procedure is the loss of pattern information hidden in the time series. To overcome this limitation, Pham used the Higuchi’s fractal dimension (HFD) [36] and proposed a new multiscale analysis [37]. The time-shift procedure illustrates the fractal dimension of a signal. This method has been recently used with entropy and cross-entropy measures [17,37,38,39]. HFD shows stable numerical results for stationary, non-stationary, deterministic, and stochastic time series [40]. For a monovariate discrete signal x of length *N*, the β time-shift signal $y_{\beta }^{\left(\tau \right)}$ is calculated as:

(33) $$y_{\beta }^{\left(\tau \right)}=\left(x_{\beta },x_{\beta +\tau },\ldots ,x_{\beta +\lfloor \frac{N-\beta }{\tau }\rfloor \tau }\right).$$

For each time scale τ, β time-shift time series are computed (β=1,2,…,τ). An illustration of the time-shift procedure is presented in Figure 1B.

### 3.3. Composite Coarse-Graining Procedure

The coarse-graining procedure, introduced by Costa et al. [14], increases the variance of estimated entropy values at large scale. To overcome this limitation, by Wu et al. introduced in 2013 a composite coarse-graining procedure [35]. This method has been used with entropy and cross-entropy measures [16,32]. For a monovariate discrete signal x of length *N*, the *k*-th composite coarse-grained time series $y_{k}^{\left(\tau \right)}$ is computed as:

(34) $$y_{k,j}=\frac{1}{\tau }\sum_{i=(j-1)\tau +k}^{j\tau +k-1}x_{i},$$

where $1⩽j⩽\frac{N}{\tau }$. For each time scale τ, *k* composite coarse-grained time series are computed (1⩽k⩽τ). An illustration of the composite coarse-graining procedure is presented in Figure 1C.

## 4. Multiscale Cross-Entropy Methods

### 4.1. Generalization

Multiscale cross-entropy (MCE) methods consist of applying a cross-entropy measure for each scale factor obtained by a specific procedure. For each scale factor τ, MCE is computed as:

(35) $$MCE\left(X^{\left(\tau \right)},Y^{\left(\tau \right)}\right)=\frac{1}{k}\sum_{\beta =1}^{k}crossEn\left(X_{\beta }^{\left(\tau \right)},Y_{\beta }^{\left(\tau \right)}\right),$$

where $X^{\left(\tau \right)}$ and $Y^{\left(\tau \right)}$ are computed with a multiscale procedure (see Section 3), *k* is the number of time series that are generated by the multiscale procedure (k=1 for the coarse-graining procedure and k=τ for the time-shift and the composite coarse-graining procedures), and crossEn is the cross-entropy method used (see Section 2). Table 2 shows the multiscale cross-entropy methods that can be generalized with Equation (35). Before the computation of MCE, a pre-treatment can be performed. For example, the asymetric multiscale cross-SampEn (AMCSE) method [33] decomposes each signal into two, one for the positive trends and the other for the negative trends, before applying a coarse-graining procedure and cross-SampEn.

### 4.2. Particular Cases

Some multiscale cross-entropy methods cannot follow the generalization previously introduced. In this part we detail three particular methods, in chronological order: The adaptive multiscale cross-SampEn, the refined composite multiscale cross-SampEn, and the percussion entropy.

#### 4.2.1. Adaptive Multiscale Cross-Sample Entropy

The adaptive multiscale cross-sample entropy (AMCSE), introduced by Hu and Liang in 2011, assesses the nonlinear interdependency between different visual cortical areas [41]. The method uses the multivariate empirical mode decomposition (MEMD), introduced by Rehman and Mandic [42], to decompose two time series into intrinsic mode functions (IMFs) that represent the oscillation mode embedded in the data. For two time series u and v, AMCSE is calculated with the following three steps algorithm:

1. The MEMD on u and v is performed to obtain *N* IMFs;
2. The scales of data are computed in two directions, fine-to-coarse $S_{f2c}^{\tau }$ and coarse-to-fine $S_{c2f}^{\tau }$, with the following two equations:
  (36) $$S_{f2c}^{\tau }=\sum_{i=\tau }^{N}{\mathrm{IMF}}_{i},\;\left(\tau ⩽N\right),$$
  (37) $$S_{c2f}^{\tau }=\sum_{i=1}^{N+1-\tau }{\mathrm{IMF}}_{i},\;\left(\tau ⩽N\right).$$
  The two directions can be used separately or used in tandem to reveal the underlying dynamics of complex time series;
3. For each scale factor τ, the cross-SampEn (see Section 2.2.1) is applied between the two scales of data ($S_{f2c}^{\tau }$ and $S_{c2f}^{\tau }$) extracted from vectors u and v.

#### 4.2.2. Refined Composite Multiscale Cross-Sample Entropy

Yin et al. introduced in 2016 the composite multiscale cross-sample entropy (CMCSE) that follows the generalization (see Section 4.1), where the composite coarse-graining procedure and cross-SampEn are used [16]. The main disadvantage of this method is that cross-SampEn generates some undefined values when the number of matched sample is zero. To overcome this limitation, Yin et al. introduced the refined CMCSE (RCMCSE). This method leads to better results with short time series. For two times series u and v of length *N*, RCMSE is computed with the following three steps algorithm:

1. Coarse-grained time series are obtained with the composite coarse-graining procedure detailed in Section 3.3;
2. For a scale factor τ, the number of matched vector pairs, $n_{k,\tau }^{m}$ and $n_{k,\tau }^{m+1}$, are calculated for all coarse-grained vectorsl
3. For each scale factor τ, RCMCSE is computed as:
  (38) $$\mathrm{RCMCSE}\left(u,v,\tau ,m,r\right)=-\ln\frac{\sum_{k=1}^{\tau }n_{k,\tau }^{m+1}}{\sum_{k=1}^{\tau }n_{k,\tau }^{m}},$$
  where *m* is the dimension and of the matched vector pairs and *r* is the distance tolerance for the matched vector pairs.

#### 4.2.3. Percussion Entropy

Wu et al. introduced, in 2013, the multiscale small-scale entropy index (${\mathrm{MEI}}_{SS}$) that is obtained by summing the values of entropy for the first five scale factors [43]. Percussion entropy, introduced by Wei et al. in 2019, allows one to quantify a percussion entropy index (PEI) [18]. The method has been introduced to assess baroreflex sensitivity. PEI compares the similarity in tendency of change between two time-series. This index has been compared to ${\mathrm{MEI}}_{SS}$. For two time series u and v of length *N*, PEI is computed with the following three steps algorithm:

1. A binary transformation of u and v is used to obtain $x=\{x_{1},x_{2},\ldots ,x_{N-1}\}$ and $y=\{y_{1},y_{2},\ldots ,y_{N-1}\}$:
  (39) $$x_{i}=\left\{\begin{array}{cc} 0 & u(i+1)⩽u(i)\\ 1 & u(i+1)>u(i) \end{array}\right.\;y_{i}=\left\{\begin{array}{cc} 0 & v(i+1)⩽v(i)\\ 1 & v(i+1)>v(i) \end{array}\right.;$$
2. The percussion rate for each scale factor τ is computed as:
  (40) $$P_{\tau }^{m}=\frac{1}{n-m-\tau +1}\sum_{i=1}^{n-m-\tau +1}\mathrm{count}\left(i\right),$$
  where *m* is the embedded dimension vectors and count(i) represents the match number between $A\left(i\right)=\left\{x_{i},x_{i+1},\ldots ,x_{i+m-1}\right\}$ and $B\left(i+\tau \right)=\left\{y_{i+\tau },y_{i+\tau +1},\ldots ,y_{i+\tau +m-1}\right\}$;
3. PEI is calculated as:
  (41) $$\mathrm{PEI}\left(m,n_{\tau }\right)=\phi^{m}-\phi^{m+1},$$
  where $\phi^{m}=\ln\sum_{\tau =1}^{n_{\tau }}P_{\tau }^{m}$ and $n_{\tau }$ is the number of scales to consider. Wei et al. [18] have chosen $n_{\tau }=5$ in accordance with ${\mathrm{MEI}}_{SS}$.

This algorithm is a generalization of the method developed by Wei et al. [18] for a specific time series, amplitudes of successive digital volume pulse signals and changes in R-R intervals of successive cardiac cycles. At the moment, it has not been used to process other kinds of signals.

## 5. Conclusions

In this review we proposed a state-of-the-art of cross-entropy measures, multiscale procedures, and multiscale cross-entropy methods. Multiscale cross-entropy methods offer other interesting perspectives for time series analysis. Furthermore, all the cross-entropy methods, detailed in this review, can be translated into multiscale cross-entropy methods with the multiscale procedures presented in this review.

## Figures and Tables

**Table 1:** Cross-entropy measures, in chronological order, that are presented in this review. Authors, year, reference, and section location are indicated for each item.

Method	Authors	Year	Ref.	Section
Cross-approximate entropy	Pincus and Singer	1996	[1]	Section 2.1.1
Cross-conditional entropy	Porta et al.	1999	[8]	Section 2.4.1
Cross-sample entropy	Richman and Moorman	2000	[2]	Section 2.2.1
Cross-fuzzy entropy	Xie et al.	2010	[3]	Section 2.4.2
Modified cross-sample entropy	Yin and Shang	2015	[4]	Section 2.2.2
Binarized cross-approximate entropy	Škorić et al.	2017	[5]	Section 2.1.2
Modified cross-sample entropy				
based on symbolic	Wu et al.	2018	[6]	Section 2.2.3
representation and similarity				
Kronecker-delta based cross-sample entropy	He et al.	2018	[7]	Section 2.2.4
Permutation based cross-sample entropy	He et al.	2018	[7]	Section 2.2.5
Cross-distribution entropy	Wang and Shang	2018	[9]	Section 2.3.1
Permutation cross-distribution entropy	He et al.	2019	[10]	Section 2.3.2
Cross-trend sample entropy	Wang et al.	2019	[23]	Section 2.2.6
Joint permutation entropy	Yin et al.	2019	[24]	Section 2.4.3

**Table 2:** Multiscale cross-entropy methods, in chronological order, that can be generalized with Equation (35). For each method, the multiscale procedure and the cross-entropy measure used and the reference are mentioned.

Method	Multiscale Procedure	Cross-entropy Measure	Reference
Multiscale cross-SampEn	Coarse-grained	cross-SampEn	Yan et al., 2009 [15]
Multiscale cross-ApEn	Coarse-grained	cross-ApEn	Wu et al., 2013 [31]
Asymetric multiscale cross-SampEn	Coarse-grained	cross-SampEn	Yin and Shang, 2015 [33]
Composite multiscale cross-SampEn	Composite coarse-grained	cross-SampEn	Yin et al., 2016 [16]
Multiscale cross-DistEn	Coarse-grained	cross-DistEn	Wang and Shang, 2018 [9]
Modified multiscale cross-SampEn			
based on symbolic	Coarse-grained	MCSEBSS	Wu et al., 2018 [6]
representation and similarity			
Modified multiscale cross-SampEn	Coarse-grained	mCSE	Castiglioni et al., 2019 [20]
Time-shift multiscale cross-SampEn	Time-shift	cross-SampEn	Jamin et al., 2019 [17]
Time-shift multiscale cross-DistEn	Time-shift	cross-DistEn	Jamin et al., 2019 [17]
Multiscale cross-trend SampEn	Coarse-grained	CTSE	Wang et al., 2019 [23]
Multiscale joint permutation entropy	Coarse-grained	JPE	Yin et al., 2019 [24]

## Supplementary Materials

The following are available online at https://www.mdpi.com/1099-4300/22/1/45/s1, Video S1: Permutation entropy–An exemple to obtain permutation vectors.
